# Phenotypic resistance not associated with knockdown mutations (*kdr*) in *Anopheles albimanus* exposed to deltamethrin in southern coastal Ecuador

**DOI:** 10.1186/s12936-023-04818-6

**Published:** 2024-01-12

**Authors:** Sebasthian Real-Jaramillo, Juan J. Bustillos, Ana L. Moncayo, Marco Neira, Leonardo Fárez, Efraín Beltrán, Sofía Ocaña-Mayorga

**Affiliations:** 1 Centro de Investigación para la Salud en América Latina, Pontificia Universidad Católica del Ecuador, Calle Pambacienda y San Pedro del Valle, Campus Nayón, 170530 Nayón, Ecuador; 2 The Cyprus Institute, Climate and Atmosphere Research Center (CARE-C), Nicosia, Cyprus; 3 Laboratorio de Referencia Intermedio de Entomología CZ707D02, Ministerio de Salud Pública de Ecuador, Machala, Ecuador; 4 Unidad Académica de Ciencias Químicas y de La Salud, Universidad Técnica de Machala, Machala, Ecuador

**Keywords:** CDC bottle assay, *Anopheles albimanus*, Resistance, *VGSC* gene, Ecuador, *kdr*, Deltamethrin

## Abstract

**Background:**

Decrease in malaria rates (e.g. incidence and cases) in Latin America maintains this region on track to achieve the goal of elimination. During the last 5 years, three countries have been certified as malaria free. However, the region fails to achieve the goal of 40% reduction on malaria rates and an increase of cases has been reported in some countries, including Ecuador. This scenario has been associated with multiple causes, such as decrease of funding to continue anti-malarial programmes and the development of insecticide resistance of the main malaria vectors. In Ecuador, official reports indicated phenotypic resistance in *Aedes aegypti* and *Anopheles albimanus* to deltamethrin and malathion, particularly in the coastal areas of Ecuador, however, information about the mechanisms of resistance have not been yet elucidated. This study aims to evaluate phenotypic response to deltamethrin and its relationship with *kdr* mutations in *An*. *albimanus* from two localities with different agricultural activities in southern coastal Ecuador.

**Methods:**

The CDC bottle assay was carried out to evaluate the phenotypic status of the mosquito’s population. Sequencing the voltage gated sodium channel gene (*VGSC*) sought knockdown mutations (*kdr*) in codons 1010, 1013 and 1014 associated with resistance.

**Results:**

Phenotypic resistance was found in Santa Rosa (63.3%) and suspected resistance in Huaquillas (82.1%); with females presenting a higher median of knockdown rate (83.7%) than males (45.6%). No statistical differences were found between the distributions of knockdown rate for the two localities (*p* = 0.6048) which indicates no influence of agricultural activity. Although phenotypic resistance was confirmed, genetic analysis demonstrate that this resistance was not related with the *kdr* mechanism of the *VGSC* gene because no mutations were found in codons 1010 and 1013, while in codon 1014, 90.6% showed the susceptible sequence (TTG) and 7.3% ambiguous nucleotides (TKK and TYG).

**Conclusions:**

These results highlighted the importance of continuous monitoring of resistance in malaria vectors in Ecuador, particularly in areas that have reported outbreaks during the last years. It is also important to elucidate the mechanism involved in the development of the resistance to PYs to propose alternative insecticides or strategies for vector control in areas where resistance is present.

**Supplementary Information:**

The online version contains supplementary material available at 10.1186/s12936-023-04818-6.

## Background

Ecuador saw a sustained decline in malaria incidence between 2000 and 2015, opening the possibility of achieving the elimination of this disease from the national territory by 2020 [1]. In fact, malaria eradication was achieved for approximately 20 years in the Ecuador-Peru border due to binational collaborative efforts for malaria control [2]. However, since 2016, the country has experienced a significant increase in the number of locally-transmitted malaria cases in both the coastal and Amazon regions of more than 1000 cases/year, challenging the elimination goal [3]. The administrative transition from the National Service of Vector-borne Diseases to the Ministry of Public Health (MoH) is perceived to have contribute to this increase in malaria incidence by both weakening early case detection, and thwarting vector control strategies that were effective in previous years [4]. The World Health Organization (WHO) has associated the changes in malaria incidence with multiple possible causes, including decreased funding to continue antimalarial programs or the monitoring of insecticide resistance [3]. The latter has been considered one of the main factors that severely complicates the control of malaria at a global scale.

The main types of insecticides used for vector control are organochlorines (OGs), carbamates (CMs), organophosphates (OPs) and pyrethroids (PYs) [5, 6]. Currently, PYs such as deltamethrin, permethrin and cypermethrin are the most widely used insecticides due to their high efficacy, low cost, high environmental stability, broad-spectrum and low toxicity to humans and the environment [7–9]. However, the constant exposure to PYs by indoor residual spraying (IRS), long-lasting insecticidal nets (LLINs) and the extensive use of insecticides in agriculture have caused the appearance of resistant insect populations in some areas [10, 11]. Resistance to PYs has been widely documented in the main African malaria vectors, such as *Anopheles arabiensis*, *Anopheles funestus* and *Anopheles gambiae *sensu lato (*s.l*.) [12–14]. In Latin America, insecticide resistance has been reported with varying magnitude in populations of some of the main malaria vectors, such as *Anopheles albimanus*, *Anopheles darlingi* and *Anopheles nuneztovari* [15–18].

Resistance to PYs is has been associated with a target-site insensitivity mechanism known as knockdown resistance (*kdr*). This mechanism involves highly specific genetic changes, such as single nucleotide polymorphisms (SNPs) or substitutions in codons 1014, 1013, 1010 and 1575 of the voltage-gated sodium channel gene (*VGSC*). Mutations that confer *kdr* resistance have been reported in codons 1010 and 1013 in *Anopheles* species from Africa and Asia. The most common type of mutations have been reported in codon 1014 and generate an amino acid change from leucine to serine (L1014S), cysteine (L1014C), phenylalanine (L1014F), or tryptophan (L1014W), thereby severely decreasing the interaction between cellular chemical receptors and insecticides [19].

The presence of resistant *kdr* alleles has been reported in populations of *An. albimanus* in Central America (México, Nicaragua and Costa Rica) [20]. In South America (Colombia and Peru), resistance to PYs in *An. albimanus* populations is somewhat less frequent, and does not pose an immediate threat to malaria control [18, 21].

In Ecuador, deltamethrin (a member of the PYs group) and malathion (a member of the OPs group) are the most commonly used insecticides for vector control [22]. Since 2018, the WHO, the Ecuadorian MoH and independent researchers have reported phenotypic resistance to both types of insecticides in *Aedes aegypti* and *An. albimanus* from the coastal areas of the country [23, 24]. However, in *An. albimanus*, the genetic traits conferring resistance have not been evaluated. As an initial step in assessing deltamethrin resistance in *Anopheles* populations in Ecuador, an evaluation was conducted on the presence of phenotypic and genotypic resistance linked to mutations in the *VGSC* gene in *An*. *albimanus* populations from southern coastal region. Furthermore, to evaluate the potential impact of land use on insecticide resistance frequency, this study compared *An. albimanus* populations from two localities with varying degrees of agricultural activity.

## Methods

### Study area

**Fig. 1:**
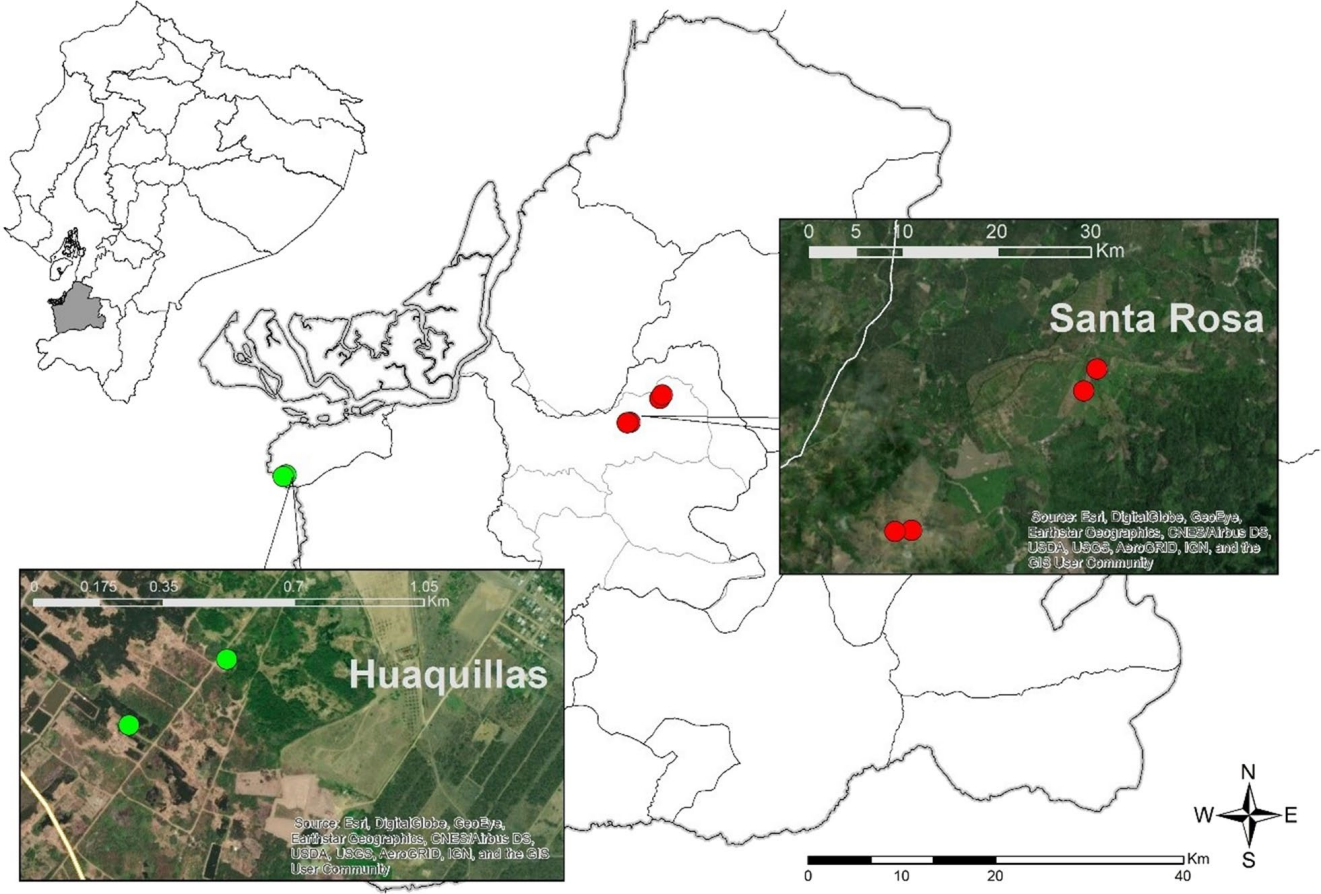
Geographical location of the collection sites in southern Ecuador (El Oro province). The red circular points showed the sampling sites from Santa Rosa (high agricultural activity) and the green points from Huaquillas (low agricultural activity)

This study was carried out in the malaria-endemic province of El Oro. This coastal province is located in the south-west region of Ecuador, and shares a border with Peru (Fig. 1). The economy of El Oro province is based on agriculture (particularly banana production), livestock and trade. During the 1980s and early 2000s, this province presented a high incidence of malaria cases; since then, a constant decline was observed, until 2011 it was deemed to be free of local malaria transmission [2]. Between 2017 and 2021, only 62 cases were reported comprising three cases of with *P. falciparum* infection and 59 cases with *P. vivax* infection [25–29].

Specimens for this study were collected in the counties of Santa Rosa and Huaquillas. Santa Rosa is one El Oro’s counties with the largest surface area dedicated to agriculture, fisheries and animal husbandry (62.68%). About 16% of the land is used for banana and cacao plantations [30]. Approximately 30% of the county’s economically-active population is involved in these activities [30]. Average precipitation during the rainy seasons (February–May and October–November) fluctuates between 500 and 2000 mm, while in the dry season (January–April) < 500 mm. Temperature in the area fluctuates from 18 to 26 °C [30]. Information regarding the use of insecticides in agriculture is limited: however, there are reports of their extensive use in areas with high agricultural activity in the province [24, 31]. In contrast, Huaquillas is a county that borders with Peru. The main economic activity is commercial trade (both formal and informal) while agriculture only contributes with 3.43% of the economic productive component [32]. Average precipitation varies from 125 to 250 mm during the rainy season (December-May) to < 20 mm during the dry season (July–December). Temperature fluctuates between 20.7 and 31 °C [32].

### Collection of immature stages and breeding

Immature stages (larvae and pupae) of *An. albimanus* were collected from March to August 2019 from temporal water ponds. In Santa Rosa, collections were carried out in livestock farms near banana plantations, while in Huaquillas specimens were collected from holes in the ground used for artisanal brick production (Fig. 1). Collections were performed using standard larval dippers (10 dips/m^2^).

Collected samples were transported live to the Intermediate Reference Laboratory CZ7 07D02 of the MoH, located in the city of Machala. Because this laboratory is located on an area where *An. albimanus* is endemic, environmental conditions were monitored but not regulated throughout the rearing process. Average environmental conditions in the area during this study were 29 ± 3 °C temperature, 80 ± 10% relative humidity.

To rear the larvae, standard guidelines for larval culture were adhere (https://www.beiresources.org/Publications/MethodsinAnophelesResearch.aspx). Larvae were placed in ceramic-coated steel trays (23 cm × 30 cm) in a density of one larva (L3/L4 stage) per ml of natural water (level 0.5 to 1 cm depth), and were fed with fish food (Levein, Quito, Ecuador). Pupae were picked daily and placed in cardboard cups covered with polyester mesh. Emerging adults were transferred to small plastic vials, identified as *An. albimanus* at the morphological level by the presence of pale-scales in the hind tarsomere 3 and 4, and the hind tarsomere 5 with a basal dark-scale [33], separated by sex and were fed with a 10% sucrose solution ad libitum.

### Phenotypic resistance by CDC bioassays

Monitoring of resistance to deltamethrin in the sampled *An*. *albimanus* populations was performed following the protocol for bottle bioassays issued by the CDC [34]. The efficiency of the deltamethrin stock technical grade (Sigma-Aldrich, Missouri, USA) used in this study was previously established in a bioassay with a susceptible strain of *Ae.aegypti* (Rockefeller strain) at a diagnosis dose of 10 µg/ml and time of 30 min.

The standardization of the CDC bottle bioassay to determine a specific diagnostic dose and diagnostic time was not feasible due to the lack of known susceptible *An*. *albimanus* strain. Thus, bioassays were conducted with the diagnostic dose (12.5 µg/ml) and time (30 min) established by CDC in susceptible *Anopheles* populations [34]. Each bioassay consisted of one control group and one to four experimental replicates, depending on specimen availability. Each assay bottle (250 ml Wheaton bottles with scroll caps) was previously impregnated with either 1 ml of deltamethrin solution (for experimental groups) or 1 ml ethanol (for control groups). Ten to 25 mosquitoes, aged 2–5 days old, were introduced in each bottle using a mouth aspirator. Due to the limited number of emerging adults, some bioassays were carried out with specimens of both sexes; in these cases, males and females were always assayed in separate bottles.

After setting up each bioassay, knockdown individuals (mosquitoes that cannot stand and that slide along the curve of the test bottle) were counted every 15 min, for a total period of 120 min. An additional counting, to estimate the mortality rate, was performed at 24 h (1440 min). After the assay, each mosquito was individually stored in a 0.6 ml microcentrifuge tube at − 20 °C for molecular analysis.

### Statistical analysis

Following CDC guidelines, each bioassay was considered valid if the mortality rate in the control bottle was up to 3% after 120 min. Assays with mortality above this threshold in the control group were not included in the analysis. If the mortality in the control bottle was between 3 and 10%, Abbott’s formula was used to correct results. A mosquito population was considered susceptible with a knockdown rate of 98% to 100%, resistance that must be confirmed from 80 to 97% and resistance if knockdown was < 80% [34].

Shapiro–Wilk test was calculated to evaluate the normality of the data and median was selected to represent the data. The non-parametric Mann Whitney test was used to compare distributions of knockdown rate between localities and sex at 30 min (diagnostic time), and to compare mortality rate at 120 min and 24 h. All analyses were performed using the STATA software (v. 15.0) [35] with a significance level for decision-making of p < 0.05. Data of bioassays are detailed in the Additional file 1: Table S1.

### Knockdown resistance (*kdr*) genotyping

A sub-sample of 50% of the mosquitoes (n = 115) used in each bioassay was selected for molecular analysis (Huaquillas n = 58, Santa Rosa n = 57). DNA extraction was carried out with a DNeasy kit (Qiagen, USA) or DNAzol (ThermoFisher, USA), following the manufacturer procedures. A 225 bp segment of the *VGSC* gene was amplified in a 25 µl reaction mixture containing 1X GoTaq^®^ Colorless Master Mix (Promega, USA) with 1.5 µM MgCl_2_, 0.2 mM dNTPs, 2.5 µM of each primer AAKDRF2 (5′-CAT TCA TTT ATG ATT GTG TTT CGT G-′3) and AAKDRR (5′-GCA ANG CTA AGA ANA GRT TNA G ′3) and 10 to 50 µg of genomic DNA [20]. This segment contains codons 1010, 1013 and 1014, and codifies for most amino acids of the *VGSC*’s sixth segment of domain II, which is critical for interaction with PYs [19, 36].

Amplification was performed using a SimpliAmp Thermocycler (Applied Biosystems, USA). Amplification conditions included an initial denaturation at 95 °C for 3 min, followed by 40 cycles at 95 °C for 45 s, 51.5 °C for 45 s and 72 °C for 1 min, and a final extension step at 72 °C for 5 min [20]. Amplification products were visualized in a 1.5% agarose gel stained with SYBR® Safe (Invitrogen, USA). Products were sequenced using a commercial service (Macrogen Inc., Korea).

Forward and reverse sequences of each sample were manually curated using MEGA11:Molecular Evolutionary Genetics Analysis version 11 [37]. Consensus alignments were established and chromatograms were manually analysed to determine the codon composition at positions 1010, 1013 and 1014. Finally, a complete alignment with all the sequences was performed, together with reference sequences of *An. albimanus* from Colombia and Guatemala (GenBank accessions MN087505 and KF137581.1, respectively). Sequence data are shown in Additional file 2: Table S2.

## Results

### Phenotypic resistance

**Fig. 2:**
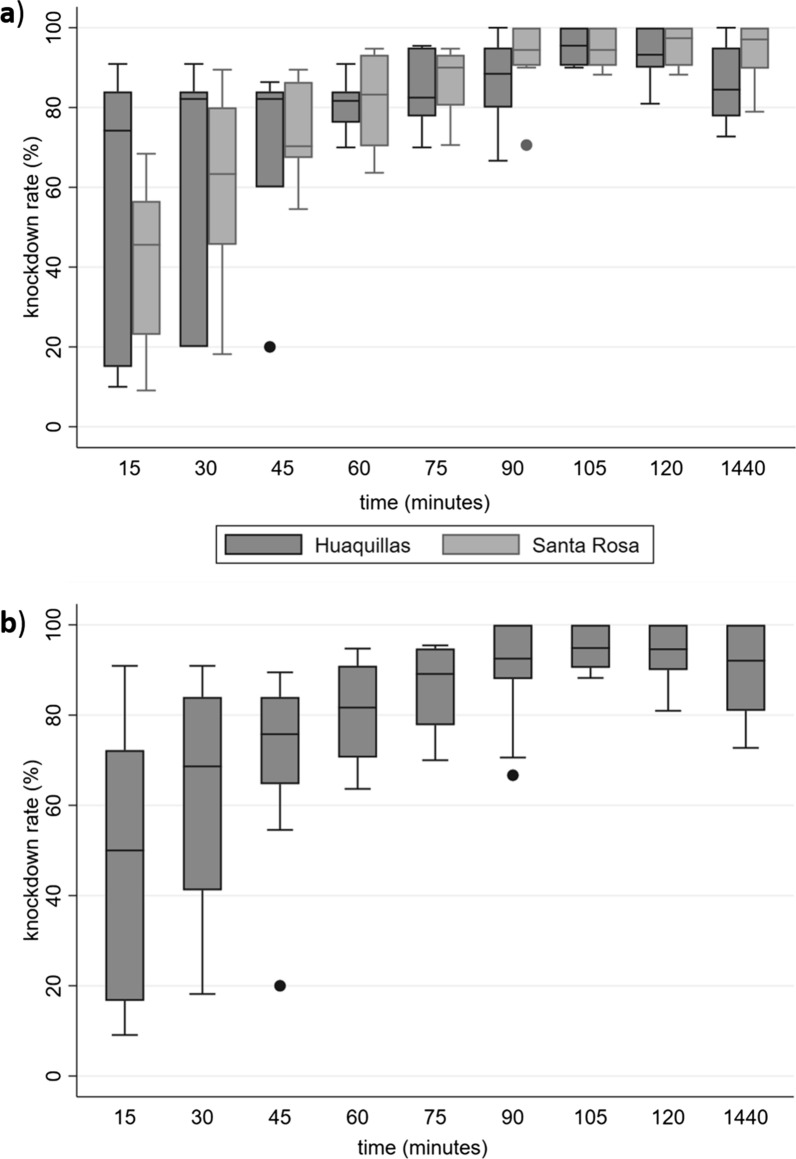
Median knockdown rate of *An. albimanus* exposed to deltamethrin at the diagnostic dose of 12.5 µg/ml. Diagnostic time was considered at 30 min. Error bars represented the maximum and minimum values. **a** Comparison of the distribution of knockdown rates by locality and **b** Total median knockdown rate

Bioassay analysis was conducted on a total of 231 *An. albimanus* specimens, with 116 from Huaquillas and 115 from Santa Rosa (Table 1). Data from five bioassays were excluded due to the absence of control bottles, control bottle mortality rates exceeding 10%, and a mix of males and females in the same experiment bottle. Knockdown rate was determined based on the median, according with the data distribution (Shapiro Wilk test, *p* = 0.03795). Mosquitoes from Santa Rosa presented phenotypic resistance with a knockdown rate of 63.3% (IQR 45.6–80.0), while in Huaquillas, suspected resistance was found with 82.1% knockdown rate (IQR 20.0–84.0). No statistical differences were found between the distributions of knockdown rate for the two localities (*p* = 0.6048) (Table 1 and Fig. 2a, b).

**Table 1:** Knockdown median proportion for *An. albimanus* in Huaquillas (HQ) and Santa Rosa (SR) in El Oro province, Ecuador.

Huaquillas (HQ)				Santa Rosa (SR)			
Time (minutes)	# Knockdown	# Non- affected	Kd %	# Knockdown	# Non- affected	Kd %	*p*-value
0	0	116	0.00	0	115	0.00	
15	74	42	74.2	50	65	45.6	
**30**	79	37	**82.1**	74	41	**63.3**	0.06048
45	82	34	82.1	87	28	70.3	
60	95	21	81.7	95	20	83.2	
75	99	17	82.5	100	15	90.0	
90	101	15	88.4	106	9	94.4	
105	111	5	95.5	109	6	94.4	
120	108	8	93.2	110	5	97.4	
1440	98	18	84.5	107	8	97.1	

Bold values corresponded to the median knockdown rate obtained at 30 minutes which constitutes the diagnosis time to determine the state of susceptibility or resistance of the population

Number of individuals counted as knockdown and alive (non-affected) by time and locality. In bold, the knockdown percentage at diagnostic time (30 min) and the associated *p* value by Wilcoxon test

Kd%: median knockdown rate

The analysis included an examination of the influence of sex on the oucomes. A total of 147 females (63.63% of total) and 84 males (36.36% of total) were used in the bioassays. Females presented a higher median knockdown rate (83.7%, IQR 73.8–89.5) than males (45.6%, IQR 20.0–60.0) (*p* = 0.0278), however no significant differences were found within localities (Huaquillas, *p* = 0.0603; Santa Rosa, *p* = 0.3836) (Table 2).

**Table 2:** Number of individuals by sex and knockdown median proportion at 30 min for *An. albimanus* from Huaquillas (HQ) and Santa Rosa (SR) in southern coastal Ecuador

	Female		Male		
	No. individuals (%)	Kd %	No. individuals (%)	Kd %	*p*-value
HQ	86 (74.13)	83.7	30 (25.86)	20.0	0.0603
SR	61 (53.04)	78.1	54 (46.95)	55.0	0.3836
HQ + SR	147 (63.63)	83.7	84 (36.36)	45.6	0.0278

Kd%: median knockdown rate, (%) proportion of individuals of each sex per locality

Comparison of knockdown rates was conducted at specific intervals: 30 min (diagnostic time), 120 min (the end of the experiment according to the protocol) and 24 h (additional record). Statistical analysis indicated significant differences when comparing knockdown rates at 30 min versus 120 min (*p* < 0.001) and versus 24 h (*p* = 0.0016), regardless the locality (Fig. 2a). Additionally, an observed increase of 13.5% and 2.9% in knockdown rate occurred between 30 and 120 min and 24 h, respectively. No statistical differences were found when comparing knockdown rate at 120 min and 24 h, regardless the locality (*p* = 0.2859) (Fig. 2a).

### Knockdown resistance (*kdr*) genotyping

**Fig. 3:**
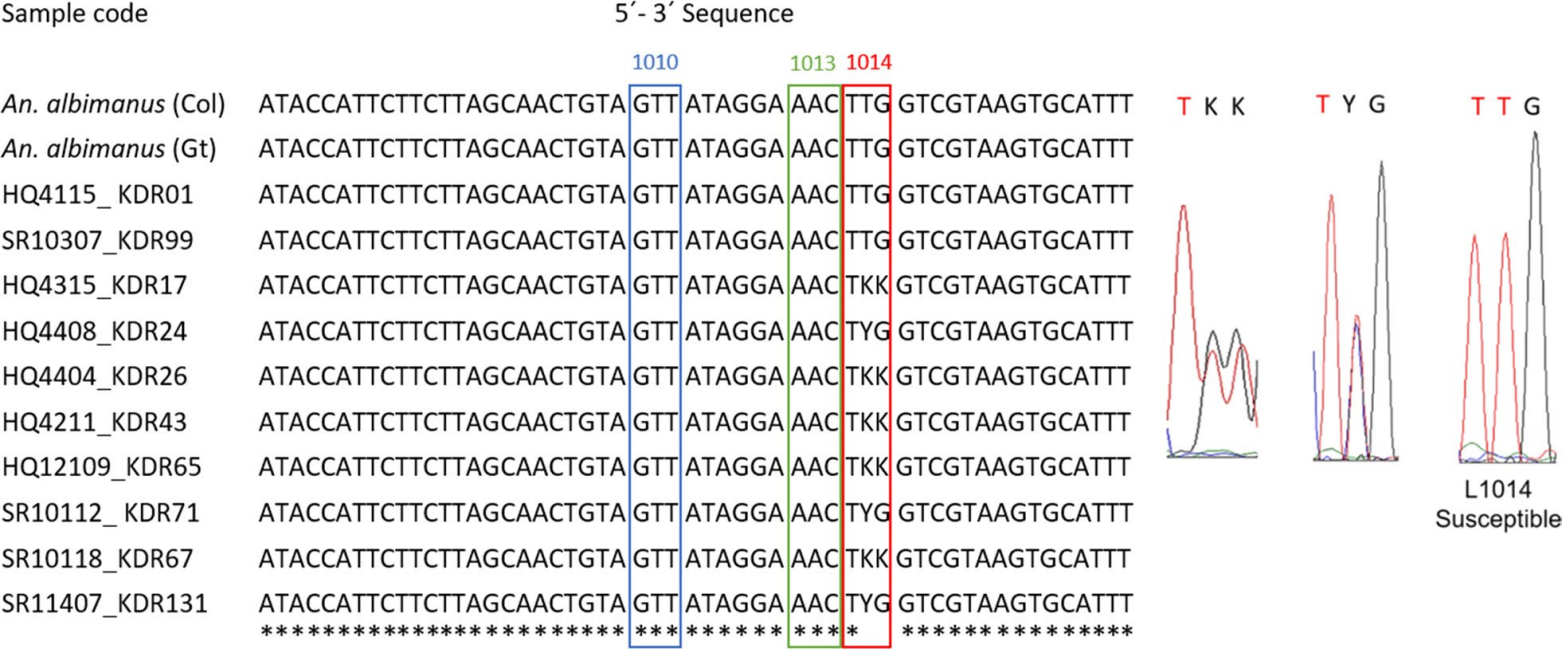
Alignment of a region of the *VGSC* gene of *An. albimanus*. Susceptible sequence (GTT) was detected for codon 1010 that codifies valine (V110) (blue box) and codon 1013 (AAC) that codifies asparagine (N113) (green box). Codon 1014 presented the susceptible sequence (TTG) that codifies leucine (red box) with the exception of certain individuals from Huaquillas (HQ) and Santa Rosa (SR) that presented ambiguous nucleotides G/T (TKK) and C/T (TYG). Alignment was performed by comparison with GenBank sequences of *An. albimanus* from Colombia (Col) (MN087505) and Guatemala (Gt) (KF137581.1). Identical positions are indicated with an asterisk

The analysis of the mutations in codons 1010, 1013 and 1014 of the *VSCG* gene was performed in 109 of the 115 sequenced samples (53 specimens from Huaquillas, 56 specimens from Santa Rosa). Six samples (5.2%) presented non-legible chromatograms and were excluded from the analysis. Only susceptible sequence (i.e. wild-type) for codons 1010 (GTT) and 1013 (AAC) were found in all samples. The susceptible sequence for codon 1014 (TTG, coding for leucine) was present in 90.6% (n = 48) of samples from Huaquillas and in 94.6% (n = 53) of samples from Santa Rosa. Ambiguous nucleotide for G/T (TKK) were found in 7.5% (n = 4) and 1.78% (n = 1) of the samples from Huaquillas and Santa Rosa, respectively. While the C/T (TYG) were found in 1.9% (n = 1) samples from Huaquillas and 3.6% (n = 2) from Santa Rosa. The codon sequences reported as confering resistance were not observed in a homozygous state in the experimental specimens (Fig. 3).

## Discussion

This study reveal the presence of phenotypic resistance to deltamethrin in *An. albimanus* populations from southern Ecuador. However, this resistance is evidenced although it was not related with *kdr* mutations, suggesting the existence of alternative resistance mechanisms in the area.

Despite the recent success of some South and CentralAmerican countries in eradicating malaria (Argentina, El Salvador and Paraguay have been certified as malaria free since 2018) [3], the elimination goal in the Americas is currently at risk. The region has failed to achieve the goal of 40% reduction on malaria rates initially set by the WHO [3], and several countries (including Honduras, Panamá, Ecuador and Bolivia) have actually reported recent increases in malaria incidence [3]. 

One of the fundamental strategies advocated by the WHO for an effective response to outbreaks is the monitoring of insecticide resistance [3]. Of the 88 malaria endemic countries, 78 have reported instances of resistance to at least one category of insecticides within their malaria vectors. The prevalence of resistance to PYs and OGs is widespread and cause alarm [3]. In the Americas, a majority of countries have actively reported plans for insecticide monitoring and management [3], underlining the pivotal role of such monitoring as an integral part of insecticide-based interventions to prevent outbreaks. Resistance status of the most important malaria vectors can be tracked by WHO-sponsored ‘Malaria Threats Map’ [38]. 

In Latin America, phenotypic resistance to insecticides (PY, OP and CM) has been reported in main malaria vectors, such as *An. albimanus*, *An. darlingi, An. nuneztovari* [17, 18, 20, 39–41]. However, the genetic traits associated with resistance in these populations are not well established. 

In Ecuador, the MoH and the National Institute of Public Health Research (INSPI, by its Spanish acronym) have reported *An. albimanus* populations resistant to PYs (Deltamethrin 0.05%), and OPs (Malathion 5%) by the discriminating concentration bioassay of the WHO [38]. Official reports have revealed deltamethrin resistance or probability of resistance in four provinces [38], including the southern coastal province of El Oro, where this study was carried out. In a 2019 report, *An. albimanus* from Huaquillas and Santa Rosa were resistant to deltamethrin (62% and 68.8% mortality rate, respectively) [23]; however, there is no data about the genetic traits or resistance mechanisms involved. 

Information regarding the use of pesticides and insecticides in El Oro province is limited; however, the use of pesticides in an area can be reflected in the number of cases related to the toxic effect of pesticides treated within the public health system. In 2019, in Santa Rosa, 20 such cases were reported, while Huaquillas had only four cases (https://www.ecuadorencifras.gob.ec/camas-y-egresos-hospitalarios/). Although Huaquillas does not have a large area of land for agriculture, a previous survey indicated that 18.9% of homes purchase pyrethroid insecticides to use at home [24]. While the extensive use of insecticides in agriculture and urban areas have been linked to the emergence of resistant insect populations [11, 42, 43], this trend was not evident in this study. Even though a lower knockdown rate is reported in Santa Rosa (locality with higher agricultural activity), no significant differences were detected when compared to Huaquillas (low agricultural activity). These results suggest a limited influence of the use of PYs in agriculture as selective pressure for mosquitos; however, certain limitations in this study (such as sample size and the use of males and females) need to be considered, as discussed below. 

One limitation to consider in this study was the limited availability of females for the bioassays that can lead to a variation of the reported knockdown proportions. In this study, female mosquitoes from 2–5 day old were used in the bioassays. However, the insufficient number of females hindering achieving an adequate sample size, thus males were included as well. Although bioassay protocols for other mosquitoes (i.e. *Aedes, Culex*) allow the inclusion of males and females as both contribute equally to the genes of their progeny [44], the modification in the methodology of this study could influence the reported knockdown proportion at diagnostic time as females presented higher rate than males. While there have not been reports of sex-linked mechanism for PYs resistance in *Anopheles*, an effect driven by sex should be considered as differential immune response and expression of insecticide resistant genes have been reported in other mosquitoes species such as *Culex pipiens* and *Ae. aegypti* [45, 46].

Another limitation of the study was the lack of a susceptible strain of *An. albimanus* to test the deltamethrin concentration, even though the viability of the chemical was tested in a susceptible strain of *Ae. aegypti* (Rockefeller). In this context, the recommended concentration [34] was applied and the knockdown rate was evaluated, instead of the mortality rate, during the CDC bioassay methodology. Additionally, an evaluation of the post-exposure effects of the insecticide were carried out by analyzing records at 120 min (the end of the experiment) and an additional reading at 24 hours, which is considered to reflect the mortality rate. The knockdown rates at 30 min were significantly lower than those at 120 min and 24 hours. This suggests that biological effects can occur not only during the period immediately following exposure to the pesticide (0-120 min), but also during the post-exposure period (120 min-24 hr). Furthermore, the decrease in the number of knockdown individuals between 120 min and 24 hour indicated that some specimens can recover after exposure to the diagnostic dose.This observation hints at the posible existence of alternative resistance mechanisms in these populations, such as metabolic resistance involving the production of detoxifying enzymes. Further exploration of the biological post-exposure effects is necessary [47].

At genetic level, *kdr* resistance on the *VGCS* gene has shown a strong causal relationship with DDT and PYs [48]. Studies in the main malaria vector species in Africa and Asia have reported non-synonymous mutation in at least four codons (1010, 1013, 1014 and 1575) of this gene [19]. In Central America, populations of *An. albimanus* have shown mutations in codon 1014 that conferred resistance to PY [20]. In South America (Colombia and Peru), phenotypically resistant populations of *Anopheles* have shown low frequency or absence of *kdr* mutations [19, 50]. These reports agree with the results of this study that reported susceptible codon sequences at position 1010 and 1013, and only eight samples (7.3%) showed polymorphisms in codon 1014.

Taken together, the presence of susceptible sequences on codons 1010 and 1013, as well as the low frequency of variants on codon 1014, suggest that the levels of resistance observed in the bioassays are most likely due to the existence of alternative resistance mechanisms in the study populations. These results are in agreement with other reports which suggest that the frequency of *kdr* mutations in a population is not necessarily the best predictor of phenotypic resistance, with other mechanisms (such as metabolic resistance) playing important roles [51, 52]. 

Furthermore, both the phenotypic and genetic results of this study agree with the description of *An. albimanus* as a panmictic population with high gene flow, particularly at microgeographic scales [53, 54]. However, as local and environmental characteristics may influence the variations observed in this vector species [54], further analysis of the impact of environment and interventions might be considered along the geographic distribution of *An. albimanus* in Ecuador. 

Between 2017 and 2021, malaria outbreaks in Ecuador were mainly reported in the Amazon region, where no data on pesticide resistance is available, and in the northern coastal area, where the only study available did not found resistance to deltamethrin in *An. albimanus* [38]. Phenotypic and genetic characterization of resistance to PYs in malaria vectors from endemic areas in Ecuador is an important step towards an early response to outbreaks. Monitoring will allow us to elucidate the mechanism involved in the development of the resistance to PYs, and to propose alternative insecticides or strategies for vector control in areas where resistance is present. This valuable information could provide a new path to control future outbreaks of malaria both locally and regionally.

### Supplementary Information

**Additional file 1: Table S1.** Counting of individuals alive and knockdown of five CDC bioassays carried out in An. albimanus from Huaquillas and Santa Rosa (southern coastal Ecuador) of 2–5 days old exposed to a deltamethrin dose of 12,5 mg/ml.

**Additional file 2: Table S2.** Sequence of codon 1010, 1013 and 1014 of the VSG gen in An. albimanus exposed to deltamethrin 12.5 mg/ml from Huaquillas and Santa Rosa, southern coastal Ecuador.

## Data Availability

All data generated or analysed during this study are included in this published article and its additional files.

## References

[1] WHO (2018). World malaria report 2018.

[2] Krisher LK, Krisher J, Ambuludi M, Arichabala A, Beltran-Ayala E, Navarrete P (2016). Successful malaria elimination in the Ecuador-Peru border region: epidemiology and lessons learned. Malar J.

[3] WHO (2022). World malaria report 2022.

[4] Mosquera-Romero M, Zuluaga-Idarraga L, Tobon-Castano A (2018). Challenges for the diagnosis and treatment of malaria in low transmission settings in San Lorenzo, Esmeraldas. Ecuador Malar J.

[5] Najera JA, Zaim M (2001). Insecticides for indoor residual spraying.

[6] Turner JA, Ruscoe CN, Perrior TR (2016). Discovery to development: insecticides for malaria vector control. Chimia.

[7] Liu N (2015). Insecticide resistance in mosquitoes: impact, mechanisms, and research directions. Annu Rev Entomol.

[8] Schleier JJ, Peterson RK, Lopez O, Fernandez-Bolanos JG (2011). Pyrethrins and pyrethroid insecticides. Green trends in insect control.

[9] Wirtz K, Bala S, Amann A, Elbert A (2009). A promise extended—future role of pyrethroids in agriculture. Pyrethroid Scientific Forum.

[10] Kona MP, Kamaraju R, Donnelly MJ, Bhatt RM, Nanda N, Chourasia MK (2018). Characterization and monitoring of deltamethrin-resistance in *Anopheles culicifacies* in the presence of a long-lasting insecticide-treated net intervention. Malar J.

[11] Reid MC, McKenzie FE (2016). The contribution of agricultural insecticide use to increasing insecticide resistance in African malaria vectors. Malar J.

[12] Kisinza WN, Nkya TE, Kabula B, Overgaard HJ, Massue DJ, Mageni Z (2017). Multiple insecticide resistance in Anopheles gambiae from Tanzania: a major concern for malaria vector control. Malar J.

[13] Messenger LA, Shililu J, Irish SR, Anshebo GY, Tesfaye AG, Ye-Ebiyo Y (2017). Insecticide resistance in *Anopheles arabiensis* from Ethiopia (2012–2016): a nationwide study for insecticide resistance monitoring. Malar J.

[14] Riveron JM, Ibrahim SS, Chanda E, Mzilahowa T, Cuamba N, Irving H (2014). The highly polymorphic CYP6M7 cytochrome P450 gene partners with the directionally selected CYP6P9a and CYP6P9b genes to expand the pyrethroid resistance front in the malaria vector *Anopheles funestus* in Africa. BMC Genomics.

[15] Dzul FA, Penilla RP, Rodríguez AD (2007). Susceptibilidad y mecanismos de resistencia a insecticidas en *Anopheles albimanus* del sur de la Península de Yucatán. México Salud Publica Mex.

[16] Fonseca-Gonzalez I, Cardenas R, Quinones ML, McAllister J, Brogdon WG (2009). Pyrethroid and organophosphates resistance in Anopheles (N.) nuneztovari Gabaldon populations from malaria endemic areas in Colombia. Parasitol Res.

[17] Fonseca-Gonzalez I, Quinones ML, McAllister J, Brogdon WG (2009). Mixed-function oxidases and esterases associated with cross-resistance between DDT and lambda-cyhalothrin in *Anopheles darlingi* Root 1926 populations from Colombia. Mem Inst Oswaldo Cruz.

[18] Orjuela LI, Morales JA, Ahumada ML, Rios JF, Gonzalez JJ, Yanez J (2018). Insecticide resistance and its intensity in populations of malaria vectors in Colombia. Biomed Res Int.

[19] Silva AP, Santos JM, Martins AJ (2014). Mutations in the voltage-gated sodium channel gene of anophelines and their association with resistance to pyrethroids—a review. Parasit Vectors.

[20] Lol JC, Castellanos ME, Liebman KA, Lenhart A, Pennington PM, Padilla NR (2013). Molecular evidence for historical presence of knock-down resistance in *Anopheles albimanus*, a key malaria vector in Latin America. Parasit Vectors.

[21] Vargas F, Córdova O, Alvarado A (2006). Determinación de la resistencia a insecticidas en *Aedes aegypti*, *Anopheles albimanus* y *Lutzomyia peruensis* procedentes del norte peruano. Rev Peruana Med Exp Salud Pública.

[22] Ministerio de Salud Publica del Ecuador (2015). Instructivo para la transferencia del talento humano, activos fijos y metodología técnica del SNEM a las entidades operativas desconcentradas del Ministerio de Salud Pública.

[23] Ministerio de Salud Publica del Ecuador (2019). Vigilancia de la resistencia a insecticidas Enero—Junio 2019.

[24] Ryan SJ, Mundis SJ, Aguirre A, Lippi CA, Beltran E, Heras F (2019). Seasonal and geographic variation in insecticide resistance in *Aedes aegypti* in southern Ecuador. PLoS Negl Trop Dis.

[25] Ministerio de Salud Publica del Ecuador (2017). Subsistema de Vigilancia SIVE-ALERTA, Enfermedades Transmitidas por Vectores Ecuador SE 1–52.

[26] Ministerio de Salud Publica del Ecuador (2018). Subsistema de Vigilancia SIVE-ALERTA, Enfermedades Transmitidas por Vectores Ecuador SE 1–52.

[27] Ministerio de Salud Publica del Ecuador (2019). Subsistema de Vigilancia SIVE-ALERTA, Enfermedades Transmitidas por Vectores Ecuador SE 1–52.

[28] Ministerio de Salud Publica del Ecuador (2020). Subsistema de Vigilancia SIVE-ALERTA, Enfermedades Transmitidas por Vectores Ecuador SE 1–52.

[29] Ministerio de Salud Publica del Ecuador (2021). Subsistema de Vigilancia SIVE-ALERTA, Enfermedades Transmitidas por Vectores Ecuador SE 1–52.

[30] GAD (2019). Plan de Desarrollo y Ordenamiento Territorial del Cantón Santa Rosa.

[31] Hutter HP, Poteser M, Lemmerer K, Wallner P, Kundi M, Moshammer H (2021). Health symptoms related to pesticide use in farmers and laborers of ecological and conventional banana plantations in Ecuador. Int J Environ Res Public Health.

[32] GAD (2019). Plan de Desarrollo y Ordenamiento Territorial del Cantón Huaquillas 2019–2023.

[33] Sallum MAM, Obando RG, Carrejo N, Wilkerson RC (2020). Identification keys to the *Anopheles* mosquitoes of South America (Diptera: Culicidae). I Introduction Parasit Vectors.

[34] Brogdon W, Chan A (2010). Guideline for evaluating insecticide resistance in vectors using the CDC bottle bioassay.

[35] StataCorp (2017). Stata Statistical Software Release 15.

[36] Dong K, Du Y, Rinkevich F, Nomura Y, Xu P, Wang L, Silver K, Zhorov BS (2014). Molecular biology of insect sodium channels and pyrethroid resistance. Insect Biochem Mol Biol.

[37] Tamura K, Stecher G, Kumar S (2021). MEGA11: molecular evolutionary genetics analysis version 11. Mol Biol Evol.

[38] World Health Organization. Vector insecticide resistance. Malaria Threats Map. 2022. [https://www.who.int/teams/global-malaria-programme/surveillance/malaria-threats-map]. Accessed 15 Feb 2022

[39] Penilla RP, Rodriguez AD, Hemingway J, Torres JL, Arredondo-Jimenez JI, Rodriguez MH (1998). Resistance management strategies in malaria vector mosquito control Baseline data for a large-scale field trial against *Anopheles albimanus* in Mexico. Med Vet Entomol..

[40] Cáceres L, Rovira J, García A, Torres R (2011). Determinación de la resistencia a insecticidas organofosforados, carbamatos y piretroides en tres poblaciones de *Anopheles albimanus* (Diptera: Culicidae) de Panamá. Biomedica.

[41] Liebman KA, Pinto J, Valle J, Palomino M, Vizcaino L, Brogdon W, Lenhart A (2015). Novel mutations on the ace-1 gene of the malaria vector *Anopheles albimanus* provide evidence for balancing selection in an area of high insecticide resistance in Peru. Malar J.

[42] Tepa A, Kengne-Ouafo JA, Djova VS, Tchouakui M, Mugenzi LMJ, Djouaka R (2022). Molecular drivers of multiple and elevated resistance to insecticides in a population of the malaria vector *Anopheles gambiae* in agriculture hotspot of West Cameroon. Genes.

[43] Kudom AA, Anane LN, Afoakwah R, Adokoh CK (2018). Relating high insecticide residues in larval breeding habitats in urban residential areas to the selection of pyrethroid resistance in *Anopheles gambiae s.l.* (Diptera: Culicidae) in Akim Oda. Ghana. J Med Entomol..

[44] McAllister JC, Scott M (2020). CONUS manual for evaluating insecticide resistance in mosquitoes using the CDC bottle bioassay kit.

[45] Rault LC, O'Neal ST, Johnson EJ, Anderson TD (2019). Association of age, sex, and pyrethroid resistance status on survival and cytochrome P450 gene expression in *Aedes aegypti* (L.). Pestic Biochem Physiol.

[46] Cornet S, Gandon S, Rivero A (2013). Patterns of phenoloxidase activity in insecticide resistant and susceptible mosquitoes differ between laboratory-selected and wild-caught individuals. Parasit Vectors.

[47] Owusu HF, Jancaryova D, Malone D, Muller P (2015). Comparability between insecticide resistance bioassays for mosquito vectors: time to review current methodology?. Parasit Vectors.

[48] Donnelly MJ, Corbel V, Weetman D, Wilding CS, Williamson MS, Black W (2009). Does kdr genotype predict insecticide-resistance phenotype in mosquitoes?. Trends Parasitol.

[49] Orjuela LI, Alvarez-Diaz DA, Morales JA, Grisales N, Ahumada ML, Venegas HJ (2019). Absence of knockdown mutations in pyrethroid and DDT resistant populations of the main malaria vectors in Colombia. Malar J.

[50] Mackenzie-Impoinvil L, Weedall GD, Lol JC, Pinto J, Vizcaino L, Dzuris N (2019). Contrasting patterns of gene expression indicate differing pyrethroid resistance mechanisms across the range of the New World malaria vector *Anopheles albimanus*. PLoS ONE.

[51] Bonizzoni M, Ochomo E, Dunn WA, Britton M, Afrane Y, Zhou G (2015). RNA-seq analyses of changes in the *Anopheles gambiae* transcriptome associated with resistance to pyrethroids in Kenya: identification of candidate-resistance genes and candidate-resistance SNPs. Parasit Vectors.

[52] Omotayo AI, Ande AT, Oduola AO, Adelaja OJ, Adesalu O, Jimoh TR (2022). Multiple insecticide resistance mechanisms in urban population of *Anopheles coluzzii* (Diptera: culicidae) from Lagos. South-West Nigeria Acta Trop.

[53] Gomez GF, Marquez EJ, Gutierrez LA, Conn JE, Correa MM (2014). Geometric morphometric analysis of Colombian *Anopheles albimanus* (Diptera: Culicidae) reveals significant effect of environmental factors on wing traits and presence of a metapopulation. Acta Trop.

[54] Altamiranda-Saavedra M, Naranjo-Diaz N, Conn JE, Correa MM (2023). Entomological parameters and population structure at a microgeographic scale of the main Colombian malaria vectors *Anopheles albimanus* and *Anopheles nuneztovari*. PLoS ONE.

